# Clinical features of benign paroxysmal positional vertigo in the elderly

**DOI:** 10.3389/fneur.2025.1623914

**Published:** 2025-06-16

**Authors:** Ran Zhou, Huan Wang, Jiachen Shan, Chengcheng Li, Lin Han

**Affiliations:** ^1^Department of Anesthesiology, Beijing Chao-Yang Hospital, Capital Medical University, Beijing, China; ^2^Department of Neurology, West China School of Public Health and West China Forth Hospital, Sichuan University, Chengdu, China; ^3^Department of Anesthesiology, Fuwai Hospital, Chinese Academy of Medical Science and Peking Union Medical College, Beijing, China; ^4^Department of Medical Cosmetology, Beijing Ji Shui Tan Hospital, Capital Medical University, Beijing, China; ^5^Department of Neurology, The Second Affiliated Hospital of Chongqing Medical University, Chongqing, China

**Keywords:** benign paroxysmal positional vertigo, BPPV, vertigo, balance, elderly

## Abstract

**Objectives:**

The typical age of benign paroxysmal positional vertigo (BPPV) is between 50 and 60 years. With the development of diagnostic techniques and the growth of the elderly, the number of elderly patients has been on the rise gradually. This study compared the clinical characteristics, treatments, and prognoses with patients.

**Methods:**

Patients were divided into two age groups based on age at onset of the disease: middle-aged BPPV (50–59 years) and elderly BPPV (60–80 years old). We compared clinical characteristics, treatment, prognosis, BPPV location, questionnaires between the two groups.

**Results:**

Female patients constituted a high proportion in both the middle-aged BPPV group (21, 75.0%) and the elderly BPPV group (39, 67.2%). The elderly group had significantly higher median scores in the DHI impact than the middle-aged group (24 vs. 16, *p* = 0.008). In contrast, the BBS score decreased (44 vs. 49, *p* = 0.019), and the elderly group exhibited higher fall rates (6.9% vs. 0%, *p* = 0.381) at the last follow-up. However, the elderly BPPV group had lower rates of maneuver (82.8% vs. 89.3%, *p* = 0.638). Age at onset was positively correlated with the DHI score (r = 0.316, *p* = 0.005) and negatively correlated with the BBS score (r = −0.330, *p* = 0.002).

**Conclusion:**

Compared to the middle-aged BPPV group, elderly patients with BPPV exhibited a higher DHI score, increased fall rates, lower BBS scores, and lower rate maneuver, which had a more significant negative impact on daily life.

## Introduction

Benign paroxysmal positional vertigo (BPPV), a peripheral vestibular disorder, is the most common cause of vertigo, (1) accounting for 17–42% of peripheral vertigo cases (2). BPPV is prevalent among individuals aged between 50 and 60 years; as the population ages, the number of elderly patients with BPPV is expected to rise (3). The clinical characteristics and prognoses of the middle-aged BPPV group may be different from those of the elderly BPPV group. Elderly BPPV often complain of unbalance without reporting vertigo, these complaints may be misdiagnosed and mistreated (4). Previous studies have identified sudden onset vertigo, a typical symptom of BPPV, usually accompanied by nausea, vomiting, and unbalance, as a significant predictor of falls in the elderly and one of the leading causes of accidental death in those aged > 65 years (5, 6). Meanwhile, other studies have identified age as an independent predictor of BPPV recurrence (7). Elderly BPPV have a high risk of falling, so elderly BPPV patients are afraid of recurrence and falls, which affect their mobility and social activities and affect their quality of life (8). Elderly patients are more prone to chronic comorbidities, such as cardiovascular and cerebrovascular diseases, endocrine diseases, and tumors, which may affect the effectiveness of repositioning and recurrence of the condition (9). However, most research has focused on the general population, with limited attention to elderly patients with BPPV. Therefore, this study aimed to assess differences in clinical characteristics, treatment, prognosis, BPPV location, Dizziness Handicap Inventory (DHI) scale, and Berg Balance Scale (BBS) between the two groups.

## Methods

### Study design and patients

**Figure 1:**
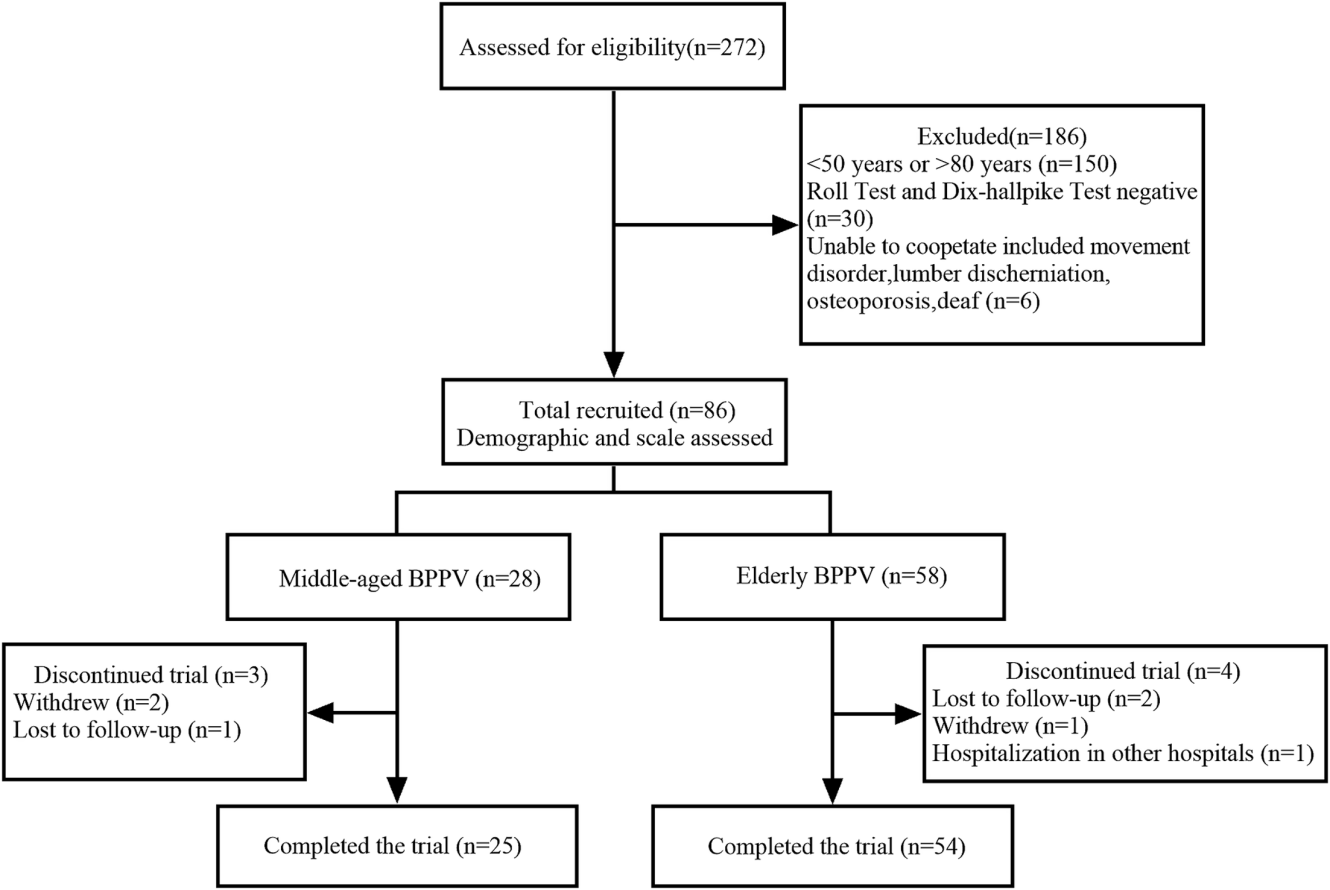
Flowchart of the study. BPPV, benign paroxysmal positional vertigo.

This study was approved by the Medical Ethics Committee of the West China School of Public Health and West China Fourth Hospital, Sichuan University (ethical approval number: HXSY-EC-V1.0). This study was conducted in accordance with the ethical standards of the Declaration of Helsinki. Informed consent was obtained from patients or their families before the study. All patients with BPPV registered on our database were recruited from the Department of Neurology, West China Fourth Hospital of Sichuan University, from February 24, 2023, to August 31, 2024. The inclusion criteria were as follows: (1) patients who presented to our department with vertigo, dizziness, or unbalance; (2) a diagnosis of BPPV according to positional nystagmus during the roll and Dix-Hallpike tests (10); and (3) age between 50 and 80 years. Exclusion criteria included: (1) diagnosis of Meniere’s disease, vestibular neuritis, persistent postural-perceptual dizziness (PPPD), delayed membranous labyrinthitis, sudden deafness with vertigo, vestibular central dizziness or mental disease; (2) negative results on the roll or Dix-Hallpike test; and (3) use of anti-anxiety and depressant medications, alcohol abuse, or inability to cooperate with the study procedures. Based on age, patients were divided into two groups: the middle-aged BPPV group (50–59 years) and the elderly BPPV group (60–80 years) (Figure 1).

### Diagnosis, treatment, and follow-up

The diagnosis of BPPV was based on the criteria established by the International Classification of Vestibular Disorders (ICVD) of the Bárány Society (1). Seventy-three patients with BPPV received the Epley, Barbeque, or Kim maneuver treatments twice, achieving a success rate of approximately 90% (11). Thirteen patients with BPPV received drug treatment. Patients were followed up once every two weeks in the first month and once a month in the second and third months. Apart from those lost to follow-up, all patients were assessed in the outpatient clinic, where they underwent re-examination with the maneuver repositioning to confirm recovery. During the follow-up, each patient was evaluated using the Dizziness Handicap Inventory (DHI) scale and Berg Balance Scale (BBS) (12, 13). The DHI assessed the impact of dizziness on life, with total scores ranging from 0 (no symptoms) to 100 (maximum symptom intensity). Scores above 60 indicated a risk of falling. The BBS evaluated balance, where higher scores represented better balance; a score of ≤20 indicated poor balance, a risk of falling, and the need for a wheelchair.

### Data collection

We collected data on age, sex, educational status, number of attacks, affected canals, clinical manifestations, treatment, and comorbidities among patients. Comorbidities included hypertension, cerebrovascular disease, heart disease, diabetes mellitus, and cancer. Additionally, we gathered information on the number of falls experienced by patients due to vertigo.

### Statistical analysis

All statistical analyses were performed using SPSS 27.0 (IBM, United States), and all figures were generated using Prism 9.5 (GraphPad Software, United States). Continuous data with normal distribution were expressed as means (standard deviations), with differences between the two groups assessed using Student’s t-test. Skewed data were reported as medians (interquartile range, IQR), and intergroup differences were evaluated using the Mann–Whitney U test. Categorical data were expressed as percentages, with differences between groups tested using chi-squared tests. Statistical significance was set at *p* < 0.05. Using G*Power 3.1.9.4 (Germany) and assuming an effect size of 0.4, we required a minimum sample of 25 patients per group to achieve a power of 0.8 and a type I error rate of 0.05.

## Results

### Demographic characteristics of patients

A total of 86 patients with BPPV were included in this study, with 28 in the middle-aged group (mean age 55.7 ± 2.8 years) and 58 in the elderly group (mean age 70.3 ± 6.1 years). No significant differences were observed between the two groups regarding education status, number of attacks, treatment, or the affected canal. Patients in the elderly BPPV group had a higher incidence of cerebrovascular disease and diabetes mellitus. Vertigo, nausea, and vomiting were more common in the middle-aged group, while unbalance was more frequently observed in the elderly group. However, these differences were not statistically significant. Detailed demographic characteristics are summarized in Tables 1, 2.

**Table 1:** Basic characteristics of the two groups.

Basic characteristics and composite scores	Middle-aged BPPV (*n* = 28)	Elderly BPPV (*n* = 58)	*p*
Sex (M/F), *N* (%)	7/21 (25.0/75.0)	19/39 (32.8/67.2)	0.617
Age, (mean ± SD, range), years	55.7 ± 2.8 (50.0–59.0)	70.3 ± 6.1 (60.0–80.0)	<0.001
Education			
Junior-middle school or below, *N* (%)	10 (35.7)	27 (46.6)	0.618
High school, *N* (%)	15 (53.6)	25 (43.1)	
Bachelor’s degree or above, *N* (%)	3 (10.7)	6 (10.3)	
Number of attacks, (mean ± SD, range)	1.7 ± 0.7 (1.0–3.0)	1.84 ± 0.9 (1.0–5.0)	0.617
Comorbidities, *N* (%)			
Hypertension	8 (28.6)	24 (41.4)	0.342
Cerebrovascular disease	2 (7.1)	16 (27.6)	0.045
Heart disease	3 (10.7)	12 (20.7)	0.253
Diabetes mellitus	0 (0)	11 (19)	0.014
Cancer	2 (7.1)	4 (6.9)	0.966
DHI, median (IQR), (baseline)	49 (36.5–64)	57 (39–72.5)	0.034
BBS, median (IQR), (baseline)	43 (36–47.8)	37.5 (29–42.5)	0.011
DHI, median (IQR), (last follow-up)	16 (0–33)	24 (20–34)	0.008
BBS, median (IQR), (last follow-up)	49 (44.5–54.5)	44 (34.8–52.3)	0.019

DHI, Dizziness Handicap Inventory; BBS, Berg Balance Scale; BPPV, benign paroxysmal positional vertigo; SD, standard deviation; IQR, interquartile range.

**Table 2:** Clinical characteristics of the two groups.

Clinical characteristics and composite scores	Middle-aged BPPV (*n* = 28)	Elderly BPPV (*n* = 58)	*p*
Clinical manifestations, *N* (%)			
Vertigo with rotational sensation	16 (57.1)	23 (39.7)	0.195
Nausea and vomiting	8 (28.6)	15 (25.9)	0.995
Unbalance	1 (3.6)	10 (17.2)	0.152
Location, *N* (%)			
PC-BPPV (right/left)	4/4 (14.3/14.3)	13/9 (22.4/15.5)	0.361
HC-BPPV (right/left)	9/4 (32.1/14.3)	15/13 (25.9/22.4)	1
AC-BPPV	7 (25)	7 (12.1)	0.226
MC-BPPV	0	1 (1.7)	1
Treatment, *N* (%)			
With Epley, Barbeque, or Kim maneuver	25 (89.3)	48 (82.8)	0.638
Without Epley, Barbeque, or Kim maneuver	3 (10.7)	10 (17.2)	
Prognosis, *N* (%)			
Fall down	0 (0)	4 (6.9)	0.381
Residual dizziness	1 (3.6)	3 (5.2)	1
Poor balance	1 (3.6)	4 (6.9)	0.885

PC, posterior canal; HC, horizontal canal; AC, anterior canal; MC, multiple canal; DHI, Dizziness Handicap Inventory; BBS, Berg Balance Scale; BPPV, benign paroxysmal positional vertigo; SD, standard deviation; IQR, interquartile range.

### Prognosis of the patients

**Figure 2:**
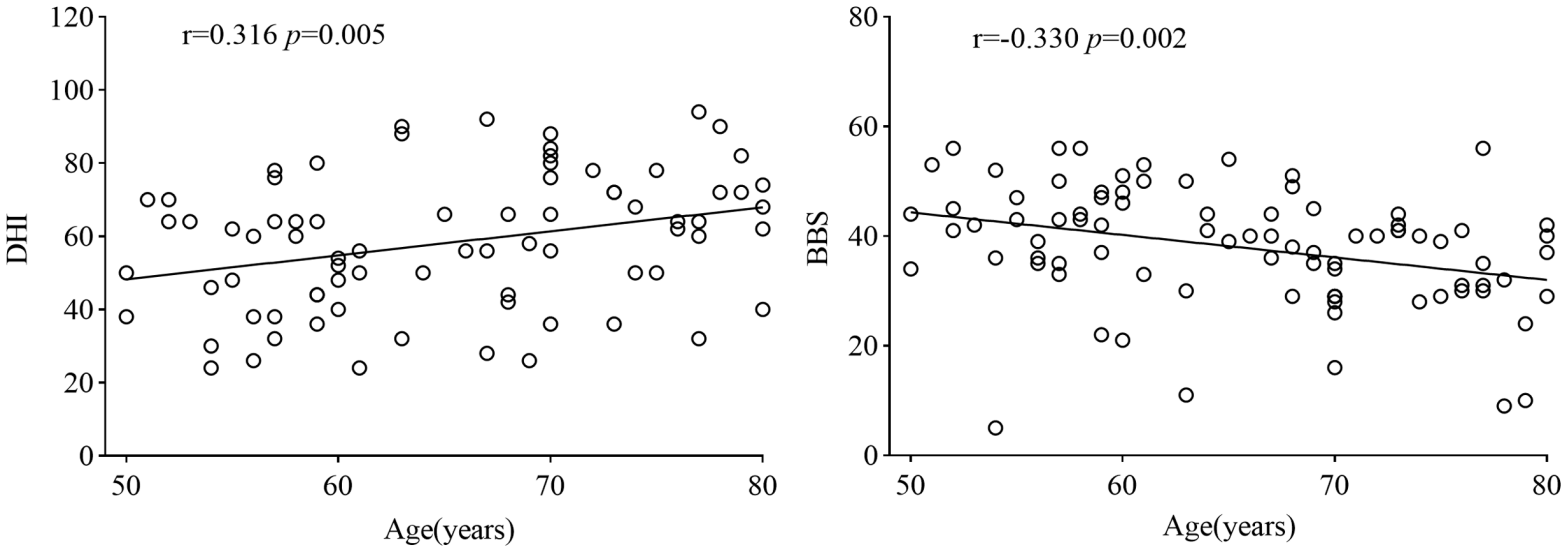
Correlation analysis of age with DHI and BBS scores. DHI, Dizziness Handicap Inventory; BBS, Berg Balance Scale.

At last, 25 patients in the middle-aged group and 54 in the elderly group completed the follow-up. The baseline median DHI score for the elderly BPPV group was significantly higher than that of the middle-aged BPPV group (57 vs. 49, *p* = 0.034; Table 1). The DHI score at the last follow-up was significantly lower than the baseline score and also higher in the elderly group (24 vs. 16, *p* = 0.008; Table 1). The baseline median score on the BBS for the elderly BPPV group was significantly lower than the middle-aged group (37.5 vs. 43, *p* = 0.011; Table 1). The BBS score at the last follow-up for the elderly group was significantly lower than that for the middle-aged group (44 vs. 49, *p* = 0.019; Table 1). The DHI score gradually increased with age (*p* = 0.005; Figure 2A), whereas the BBS score decreased (*p* = 0.002; Figure 2B). To figure out whether there is a correlation among age, the DHI and the BBS, the multiple liner regression was conducted and the results indicated that there is no linear correlation among DHI, BBS, and age (r^2^ = 0.143, DHI *p* = 0.067, BBS *p* = 0.058, Supplementary Figure 1). During the follow-up, four individuals in the elderly group experienced falls due to vertigo, warranting specific descriptions as falls are a leading cause of fractures and accidental non-disease deaths among the elderly: (1) two patients experienced vertigo while getting up, which caused them to lose their balance and fall down, with one sustaining a head hematoma without a stroke; (2) one patient fell down due to severe vertigo that made her unable to open eyes, resulting in a compression fracture of the lumbar spine that did not require surgery; and (3) one patient experienced vertigo, nausea and vomiting while turning over on the bed. She accidentally fell from the bed onto the floor.

## Discussion

In this study, we evaluated the effect of age on basic characteristics and clinical manifestation in patients with BPPV. A total of 86 patients were included and divided into the 28 middle-aged (50–59 years) BPPV group and 58 elderly (≥60 years) BPPV group. The clinical manifestations, prognosis, and impact on quality of life vary depending on the age of onset. Although the general expectation of “age-related functional decline” is well-established, our quantitative data demonstrate a striking dissociation in elderly BPPV patients: a 50% increase in vertigo severity coexists with a 10% decline in balance function, which may synergistically exacerbate fall risk (6.9% in the elderly vs. 0% in the middle-aged group). Our research supports the idea that the effects of brain aging are not singular but systematic, affecting not only the central balance system but also the peripheral balance system (4). This finding suggests that elderly BPPV is not merely a vestibular disorder but rather a window into “multisystem aging,” necessitating integrated management combining vertigo treatment and comprehensive geriatric care.

In the basic characteristics section of this study, we found that BPPV was more common in two groups of female patients, especially in perimenopausal women, which is consistent with previous reports (4). This age group has a high incidence of BPPV, which may be due to the decrease in estrogen levels and significant calcium loss in perimenopausal and postmenopausal women, leading to unstable and recurrent loss of otoliths (14).

In previous studies, PC-BPPV was the most common type (3). However, in our study, besides PC-BPPV, HC-BPPV was also higher in the elderly group than in the middle-aged and elderly group, which is consistent with Song’s research results (4). The most common reason for PC-BPPV may be that previous studies did not differentiate by age. BPPV in the elderly group usually involves multiple semicircular canals, including the posterior and horizontal canals, which may be related to the functional degradation of the utricle and saccule (15).

Compared with the middle-aged group, the elderly group showed worse balance function and more complications, especially cardiovascular disease and diabetes, which had a greater negative impact on daily life (16). This may be related to vestibular dysfunction in elderly patients, coupled with osteoporosis-related calcium loss, leading to instability and increased susceptibility to canalith displacement (17–19). Simultaneously, comorbid conditions, including vitamin D deficiency, cerebrovascular disease, and diabetes, can exacerbate this impact. Cerebrovascular disease can cause anterior inferior cerebellar artery atherosclerosis, leading to stenosis, whereas diabetes can impair microcirculation and contribute to cerebral arteriosclerosis and stenosis, affecting vestibular blood supply (9, 20).

The devastating effects of BPPV include a lack of awareness of the disease, falls, and a serious impact on the quality of life for both patients and their families. Some elderly patients may doubt the effectiveness of manual repositioning and opt for drug therapy instead. Others might fear that manual reduction could worsen their symptoms or cause fractures, leading them to refuse the procedure. In our study, 10 elderly and three middle-aged patients avoided manual repositioning. Notably, the lower canalith repositioning rate in the elderly group (82.8% vs. 89.3%, *p* = 0.638), when coupled with their more severe symptoms and functional impairments, may reflect undertreatment in clinical practice (e.g., reluctance to perform maneuvers due to comorbidities or misjudged tolerance). A 2021 meta-analysis found that the complete recovery rate in older adults is comparable to that in younger adults (3). This highlights the need to optimize therapeutic pathways for elderly BPPV, such as developing age-tailored repositioning protocols or adjunctive interventions. Therefore, promoting manual repositioning as a treatment option for elderly patients is crucial, along with raising awareness about BPPV and educating the public about the condition.

Falls, a major concern, were reported in nearly 7% of the elderly group in our study. Given the short observation period and small sample size, the actual rate of falls and associated serious outcomes, such as fractures, cerebral hemorrhage, or even death, is likely higher in real-life scenarios. Elderly patients, who often experience increased vestibular dysfunction, poor balance, and severe vertigo symptoms, may develop a fear of falling, leading them to avoid going out alone or staying home alone. This fear can place a significant burden on both elderly individuals and their families (21).

Efforts should focus on increasing awareness of BPPV among elderly patients to help them better understand the condition, alleviate their fears, and consider treatment options beyond medication. Encouraging more patients to participate in manual repositioning therapy, the most effective treatment for this condition, can improve their quality of life, reduce the occurrence of accidents, and lessen the burden on their families. Furthermore, the strong positive correlation between age and DHI and negative correlation with BBS imply diminished vestibular compensatory capacity in elderly BPPV patients, potentially explaining divergent clinical outcomes despite shared pathology (otolith displacement). These findings not only confirm the hypothesis of “greater BPPV severity in the elderly” but also illuminate underlying clinical challenges (e.g., management gaps) and research directions (e.g., mechanisms of age-related vestibular decline).

This study had several limitations. Firstly, while we calculated the sample size, the small sample, particularly with only 28 patients in the middle-aged group, may have affected the robustness of the results. There are differences between the two groups regarding gender, clinical manifestations, location, treatment, and prognosis, but they are not statistically significant, which may relate to the small sample size. Large sample validation is needed at a later stage. Secondly, the follow-up period of this study was short. Longer follow-up studies could provide a comprehensive assessment of treatment effects and patient quality of life. Future research should aim to explore long-term outcomes, including relapse rates, residual vertigo, and mental health impacts.

## Conclusion

Compared with the middle-aged BPPV group, elderly patients with BPPV had a higher DHI score, higher fall-down rates, lower BBS score, and lower Epley, Barbeque, or Kim maneuver rates, which had a greater negative impact on daily life.

## Data Availability

The original contributions presented in the study are included in the article/Supplementary material, further inquiries can be directed to the corresponding author.
