# Supplementary material for: Clinical features of benign paroxysmal positional vertigo in the elderly
Source: Front Neurol. 2025 Jun 16;16:1623914. doi: 10.3389/fneur.2025.1623914 (PMC12206830; doi:10.3389/fneur.2025.1623914)
Supplement: Supplementary file 1 [file Supplementary_file_1.DOCX]

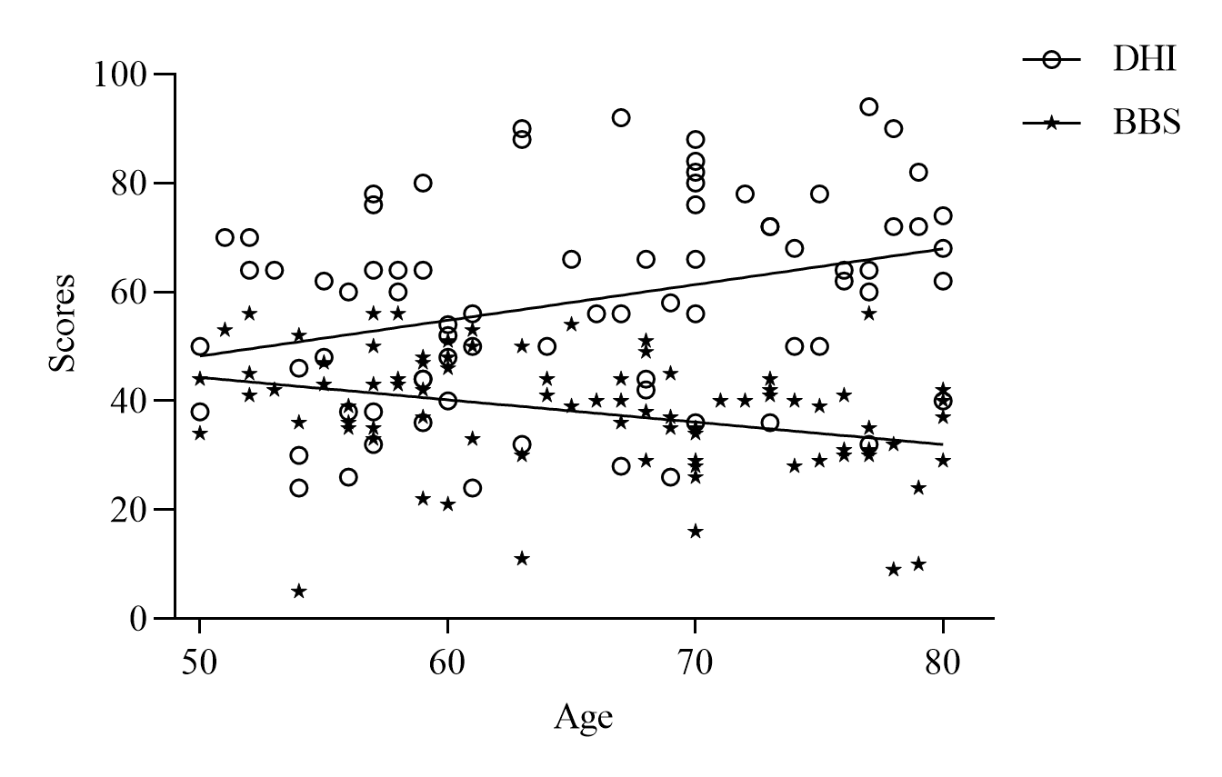


**Supplementary Figure 1**. Multiple linear regression analysis of age with DHI and BBS scores.

DHI = Dizziness Handicap Inventory, BBS = Berg Balance Scale.
